# TCIRG1 as a Novel Prognostic Biomarker Triggering Immune Infiltration in Renal Clear Cell Carcinoma: An Integrative Study of Single-Cell and Bulk Data

**DOI:** 10.1155/humu/1839494

**Published:** 2025-10-24

**Authors:** Wei Ye, Honghao Yang, Xincheng Yi, Shaoyi Zhang, Siyu Wang, Zongming Jia, Jin Zang

**Affiliations:** ^1^Department of Urology, The First Affiliated Hospital of Soochow University, Suzhou, Jiangsu, China; ^2^Department of Urology, Guanyun People's Hospital, Lianyungang, Jiangsu, China

**Keywords:** immune infiltration, kidney renal clear cell carcinoma, prognosis, TCIRG1, translational biomedical informatics

## Abstract

**Background:**

Tumor microenvironment (TME) is a significant factor regulating the malignant phenotype and drug resistance of kidney renal clear cell carcinoma (KIRC). The identification of biomarker signatures mediating immune infiltration in TME is of significance for prognostic assessment and personalized therapy of KIRC.

**Methods:**

The gene set associated with immune cell populations in KIRC TME was extracted from the single-cell dataset GSE139555 using high-dimensional weighted coexpression network analysis (hdWGCNA). The bulk data from TCGA-KIRC were integrated to screen significant signatures in KIRC prognosis through Cox regression, and a combination of 101 machine learning algorithms was compared to prioritize feature genes for the construction of a novel prognostic model. Finally, LightGBM and XGBoost algorithms identified TCIRG1 as a key model feature and a novel biomarker in KIRC for experimental characterization using western blot, immunohistochemistry, multiple immunofluorescence (mIHC), subcutaneous tumor formation in nude mice, and Transwell assays.

**Results:**

Single-cell data showed that the monocyte population varied most significantly in KIRC samples, and 150 candidate genes from monocytes were identified based on hdWGCNA. By integrating bulk TCGA-KIRC data and Cox regression, 15 prognosis-related genes were extracted as candidates for machine learning–powered training using 101 algorithm combinations, and nine genes were prioritized as feature variables to establish a prognostic model with good predictive performance on the overall survival of KIRC patients. Finally, TCIRG1 was identified as a novel biomarker signature from the prognostic model, and ultimately, by combining LightGBM and XGBoost algorithms, TCIRG1 was identified as a key characteristic signal for experimental validation and functional studies. Immunohistochemistry, cellular, and animal experiments showed that TCIRG1 expression was significantly elevated in KIRC samples, and its high expression was closely associated with adverse clinicopathological features. mIHC results demonstrated a significant positive correlation between TCIRG1 expression and immune cell infiltration in the KIRC TME, particularly with Treg cells.

**Conclusions:**

TCIRG1 was identified and validated as a novel prognostic biomarker triggering immune infiltration in KIRC. The mechanisms and translational prospects of TCIRG1 in KIRC management will be explored in future work.

## 1. Introduction

Kidney renal clear cell carcinoma (KIRC) represents a highly malignant form of urinary tract cancer, posing significant health risks [1]. In 2022, global kidney cancer cases reached 434,419, constituting 2.2% of all malignant tumor incidences, with 155,702 fatalities reported [2]. Among these cases, renal cell carcinoma (RCC) represents the primary portion (90%) of kidney cancers, primarily consisting of clear cell carcinoma (KIRC, 70%), papillary renal carcinoma (pRCC, 10%–15%), and chromophobe cell carcinoma (5%), with other subtypes being relatively uncommon. WHO statistics from 2020 reveal that 36.6% of newly diagnosed renal cancer cases occurred in Asia [3]. While early-stage kidney cancer is often treatable through surgery, advanced kidney cancer exhibits limited responsiveness to conventional chemotherapy and radiotherapy [4]. Consequently, immune checkpoint inhibitors (ICIs) and molecularly targeted therapies have become the primary treatment modalities [5]. Targeted drugs, including vascular endothelial growth factor (VEGF) inhibitors such as sunitinib and sorafenib, as well as mTOR inhibitors like temsirolimus, are extensively applied in clinical settings [6–8]; however, many patients exhibit primary or acquired resistance to drugs such as sunitinib and sorafenib within 6–15 months of treatment [9, 10]. A 2018 Phase II clinical trial led by Park et al. further demonstrated temsirolimus's limited efficacy compared to VEGF inhibitors in terms of progression-free survival and objective response rate [11]. Recent studies suggest that the unique tumor microenvironment (TME) associated with advanced renal cancer [12–15] may play a critical role in drug resistance, particularly in highly invasive renal carcinoma. The composition and activity of immune cells and factors during infiltration often vary significantly between high-risk and low-risk renal carcinomas [16–18]. This study is aimed at identifying novel immunomodulatory targets within the TME to propose alternative therapeutic strategies for advanced KIRC.

The TME is critical in modulating tumor progression [19–24], with regulatory T cells (Tregs)—a subset of T cells—demonstrating high infiltration in both inflammatory tissues and the TME of various tumors [25–27]. Studies indicate that Treg cells can suppress tumor immune reactions by modulating multiple immune cells, encompassing NK cells and B cells, through various mechanisms [28]. T cell immune regulator 1 (TCIRG1), also known as T cell immune response cDNA7 (TIRC7), has been implicated in promoting progression and metastasis in malignancies, encompassing prostate cancer [29], breast cancer, and hepatocellular carcinoma [29–31]. Recent findings reveal that TCIRG1 expression in RCC is significantly elevated compared to adjacent tissues [32, 33], with high TCIRG1 expression closely associated with poorer overall survival (OS). Additionally, TCIRG1 shows strong coinfiltration with immune-related cells, including monocytes, NK cells, CD8+ T cells, and CD4+ T cells.

In this investigation, data from The Cancer Gene Atlas (TCGA) and GEO public databases were combined to develop a new KIRC prognosis model. Using machine learning, TCIRG1 was identified as a key gene, and its expression in KIRC samples was validated through WB, multiplex immunohistochemical fluorescence (mIHC), scratch assays, and Transwell assays to assess clinical significance. Results suggest that TCIRG1 serves as a key biomarker for KIRC assessment and exhibits potential as a promising immunotherapy target for individuals with advanced KIRC in the future.

## 2. Materials and Methods

### 2.1. Dataset Collection

The datasets for all KIRC samples were sourced from the TCGA-KIRC database (https://portal.gdc.com), with gene expression verification conducted using the GEO database (https://www.ncbi.nlm.nih.gov/geo/). Statistical analysis and data visualization were performed with R software v4.0.3 and the ggplot2 package (v3.3.2). A significance threshold of *p* < 0.05 was utilized.

### 2.2. Single-Cell Analysis Identifies Distinct Cell Populations in KIRC and Extracts Associated Genes

A comprehensive search using the keyword “renal clear cell carcinoma” in the GEO database (https://www.ncbi.nlm.nih.gov/geo/) retrieved relevant data from dataset GSE139555. The R package Seurat [34, 35] was employed to generate objects, filter low-quality cells, and carry out standard preprocessing, including cell quality control and batch processing. Hypervariable genes were identified, followed by principal component analysis and UMAP for dimensionality reduction to detect cell clusters with similar gene expression patterns. The analysis revealed distinct marker genes in monocytes, T cells, B cells, and NK cells, with monocyte subsets exhibiting the most significant changes between normal and tumor groups. Weighted gene coexpression network analysis (WGCNA) was conducted, where a soft threshold was set, and a cluster dendrogram was generated to identify color-coded modules. The top 50 genes from each of the three subpopulation modules were selected, totaling 150 genes for further investigation.

### 2.3. Integrating Bulk Omics Data and Combined Machine Learning Algorithms to Screen for Key Genes and Construct a Prognostic Model

Subsequently, these 150 genes were analyzed for differential expression within the TCGA database. Within the TCGA-KIRC database, 150 genes previously identified via single-cell analysis were selected for differential expression analysis and Cox proportional hazards regression analysis. This process identified 15 core genes significantly associated with the outcome of interest. Subsequently, a protein–protein interaction (PPI) network was constructed for these 15 genes. Consensus clustering analysis was then performed based on the expression profiles of these 15 genes using the R package ConsensusClusterPlus (v1.54.0). A consensus matrix heatmap demonstrated that the clearest cluster separation was achieved at *k* = 2. Consequently, KIRC patients were stratified into two distinct molecular subgroups (designated Group A and Group B) according to their expression patterns of these core genes. Finally, prognostic disparities and differences in immune infiltration abundance between Group A and Group B were comprehensively analyzed.

### 2.4. Extracting Key Genes as Biomarkers

To evaluate patient prognosis, this study utilized the TCGA-KIRC database (*n* = 529) as the training set and the E-MTAB-1980 dataset from ArrayExpress (*n* = 101) as the validation set. The optimal StepCox[both] + RSF algorithm combination, selected from 101 machine learning algorithm combinations, was employed to identify nine core genes for constructing the prognostic model. ROC curve analysis demonstrated high AUC values for both the training and validation sets at 1-year, 3-year, and 5-year intervals, confirming the model's robust predictive performance. Additionally, a nomogram was developed by integrating clinical data to further enhance prognostic assessment.

### 2.5. Clinical Specimen Collection

From January to June 2024, eight individuals with renal cancer participated in this investigation, all of whom underwent radical nephrectomy upon admission and were diagnosed with KIRC by the Department of Pathology at the First Affiliated Hospital of Soochow University. Tumor tissue served as the experimental group, while adjacent tissue was used as the normal control. This investigation was sanctioned by the Ethics Committee of the First Affiliated Hospital of Soochow University on January 5, 2024, before the experiment (Review No.: (2024) Lunyan Approval No. 545).

### 2.6. Cell Culture, RNA Interference, and WB Experiments

The human renal tubular epithelial cell line HK-2 (RRID: CVCL_0302) and the renal clear cell carcinoma cell lines 786-O (CVCL_1051), 769-P (RRID: CVCL_1050), ACHN (RRID: CVCL_1067), and CAKi-1 (RRID: CVCL_0234) were acquired in 2023 from the Cell Bank of the Committee on Type Culture Collection of the Chinese Academy of Sciences, located in Shanghai, China. All these cell lines were cultured in 1640 medium supplemented with 10% fetal bovine serum and maintained in a humidified incubator at 37°C with 5% CO_2_. We confirmed that the cell lines used were free from contamination and accurately identified.

Transfections were performed on six-well plates at optimal cell density utilizing siRNA from Shanghai Jikai Gene Technology Co. Ltd., China, 1 day prior to the procedure. Forty-eight hours posttransfection, half of the cells were collected for ultrasonic lysis, and protein concentration was ascertained utilizing a BCA kit (Beyotime, China). For SDS-PAGE analysis, 10 *μ*L of the sample was loaded onto the gel, proteins were transferred onto a PVDF membrane, sealed with 5% skim milk at ambient temperature for 2 h, and maintained overnight with the primary antibody (Immunoway, United States) at 4°C in darkness. Subsequently, the membrane underwent incubation with a horseradish peroxidase–linked secondary antibody followed by signal visualization.

### 2.7. Subcutaneous Tumor Formation Experiment in Nude Mice

Nine healthy 6-week-old male nude mice weighing 18–22 g were selected as experimental subjects. The mice were housed in a controlled environment with constant temperature (22°C–26°C) and humidity (40%–60%), under a 12-h light/dark cycle. After 1 week of acclimatization, 769-P cells were collected at 80%–90% confluence, centrifuged to remove the supernatant, resuspended, and counted. A cell suspension was prepared, and each nude mouse was inoculated with 100 *μ*L of the suspension. The survival status of the mice and tumor growth were monitored daily. The maximum (*L*) and minimum (*W*) lengths of the tumors were measured, and tumor volume (*V*_T_) was calculated using the formula *V*_T_ = 1/2 × (*L* × *W* × *W*). When the tumor diameter reached 15–20 mm, the mice were euthanized by cervical dislocation. The tumor tissues were surgically excised, weighed, and photographed.

### 2.8. Immunohistochemistry and mIHC

Tumor tissues from the eight patients with KIRC were procured from the First Affiliated Hospital of Soochow University. Sections underwent paraffin embedding and were sectioned to 5-*μ*m thickness. Immunohistochemical staining was performed on one group, involving manual dewaxing with xylene, followed by washing with an ethanol gradient and ddH_2_O. Antigen recovery was achieved by treating the sections in sodium citrate buffer under elevated temperature and pressure conditions, incubated with H_2_O_2_, rinsed with PBS, and blocked with 3% BSA solution. Specific antibodies against DAPI, CD152, FoxP3 (Forkhead Box Protein P3), and TCIRG1 were applied for staining, with fluorescence microscopy used for histological diagnosis. Informed consent was procured from all patients, and all procedures were sanctioned by the Ethics Committee of the First Affiliated Hospital of Soochow University, adhering to established institutional guidelines and regulations.

### 2.9. Transwell Experiment

The upper chamber of the Transwell insert was coated with Matrigel (Corning, United States) at a ratio of Matrigel to serum-free medium of 1:6. Following 48 h of transfection, cells were resuspended in serum-free medium, and 5 × 10^4^ cells were introduced into the upper chamber. After 48 h, cells in the upper chamber were stabilized utilizing 4% paraformaldehyde at ambient temperature for 20 min and subsequently stained with crystal violet for 20 min. Images were acquired utilizing a Nikon T12-D-PD inverted microscope (Nikon, Japan), and migrating cell counts were assessed using ImageJ software.

### 2.10. Statistical Analysis

Statistical analyses were primarily executed with R Software Version 4.2.1 or GraphPad Prism 6. Additionally, two machine learning algorithms, LightGBM and XGBoost, were implemented in Python, with model interpretation performed using SHAP. Differences between variables were assessed using the *t*-test or one-way ANOVA as appropriate. Nonparametric tests, including the Wilcoxon test and the Kruskal–Wallis test, were applied when variance homogeneity was not met. Correlations between variables were examined utilizing Pearson's or Spearman's correlation as appropriate. All *p* values were two-sided, with *p* < 0.05 deemed statistically significant.

## 3. Results and Conclusions

### 3.1. Screening of Prognostic Factors Through Single-Cell Analysis

**Figure 1:**
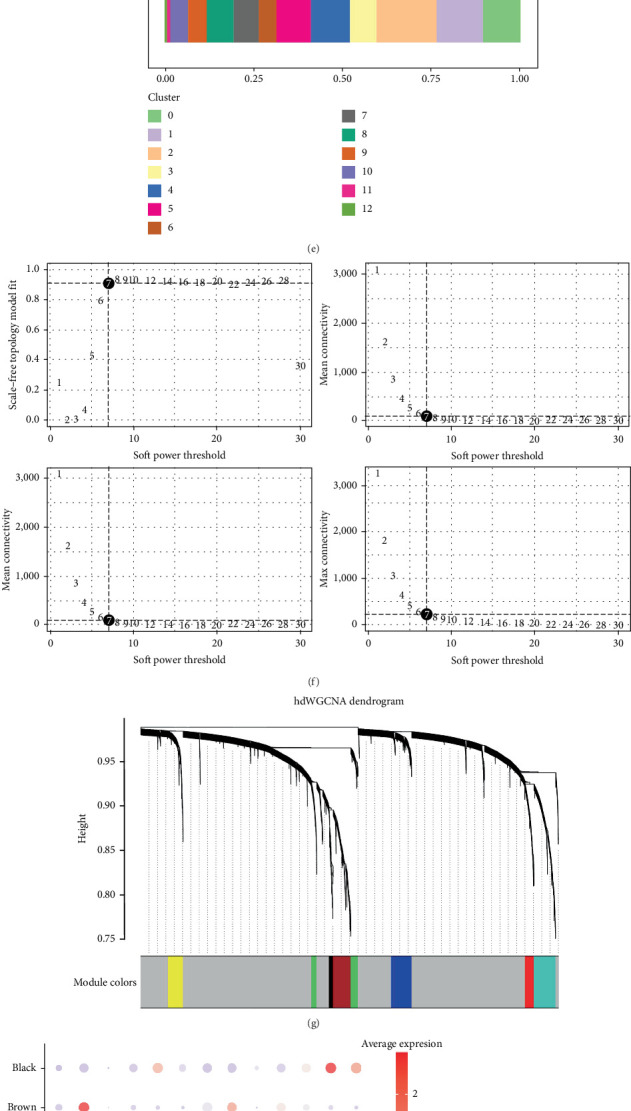
Single-cell RNA sequencing analysis identifies 15 prognostic factors. (a) UMAP plot generated from KIRC and normal tissue samples in single-cell analysis to identify the 15 prognostic factors most correlated with KIRC. (b) UMAP profile with distinct cell clusters color-coded. (c) Monocyte proportions show the greatest increase in KIRC compared to normal tissue, warranting further analysis. (d) Comparison of monocyte subset composition between KIRC and normal tissues. (e) Subpopulations 0, 3, and 6 exhibit the largest variations. (f) Selection of hdWGCNA soft threshold set at 7. (g) Dendrogram illustrating seven gene modules in distinct colors. (h) Bubble chart showing module-specific scores. (i) Heatmap of genes with significant expression differences. (j) Differential genes were subjected to univariate Cox regression analysis, identifying 15 prognostic factors. (k) Interaction network of the 15 prognostic factors.

To examine the variations in immune cell composition and proportions between KIRC and normal kidney tissue, dataset GSE139555 from the GEO database (https://www.ncbi.nlm.nih.gov/geo/) was analyzed for immune cell infiltration in KIRC. Principal component analysis, dimensionality reduction, clustering, and cell type annotation were applied to integrate the data (Figure 1a,b). Compared to normal kidney tissue, B and T cell proportions were reduced in KIRC, while NK cell proportions showed a slight increase. Monocyte subsets displayed the most notable increase (Figure 1c). This subset was further examined following the aforementioned methodology, revealing that Subsets 0, 3, and 6 exhibited the most pronounced changes between KIRC and normal kidney tissues (Figure 1d,e).

To characterize each subgroup, hdWGCNA analysis was conducted. The data were cleaned and standardized in R (Figure 1f), and a soft power threshold of 7 was determined through four analytical functions, yielding a clustering dendrogram and identifying seven gene modules (Figure 1g). Among these modules, blue, green, and red were highly expressed in Subsets 0, 3, and 6, showing a significant positive correlation trend (Figure 1h). The top 50 genes from each module were selected, resulting in 150 genes for further analysis.

To refine the KIRC-associated gene set, RNA-seq data for the aforementioned 150 genes were extracted from the TCGA-KIRC database. Following normalization and exclusion of samples with missing data, differential expression analysis and univariate Cox proportional hazards regression analysis were performed. This identified 15 genes most significantly associated with KIRC prognosis. Among these 15 genes, elevated expression of 13 genes (MYO9B, TNFAIP2, PLAUR, CXCL2, TREM1, CEBPB, UBXN11, TCIRG1, SLC11A1, RPS17, EFHD2, CD44, and CD300E) in tumor tissue exhibited a positive correlation with poor prognosis. Conversely, high expression of IQGAP2 and LRRK2 in tumor tissue demonstrated a negative correlation with poor prognosis. All associations were statistically significant (*p* < 0.001) (Figure 1i,j). Finally, a PPI network was constructed for these 15 prognostic genes (Figure 1k).

### 3.2. Immune Profiling Validation of Relevant Genes

**Figure 2:**
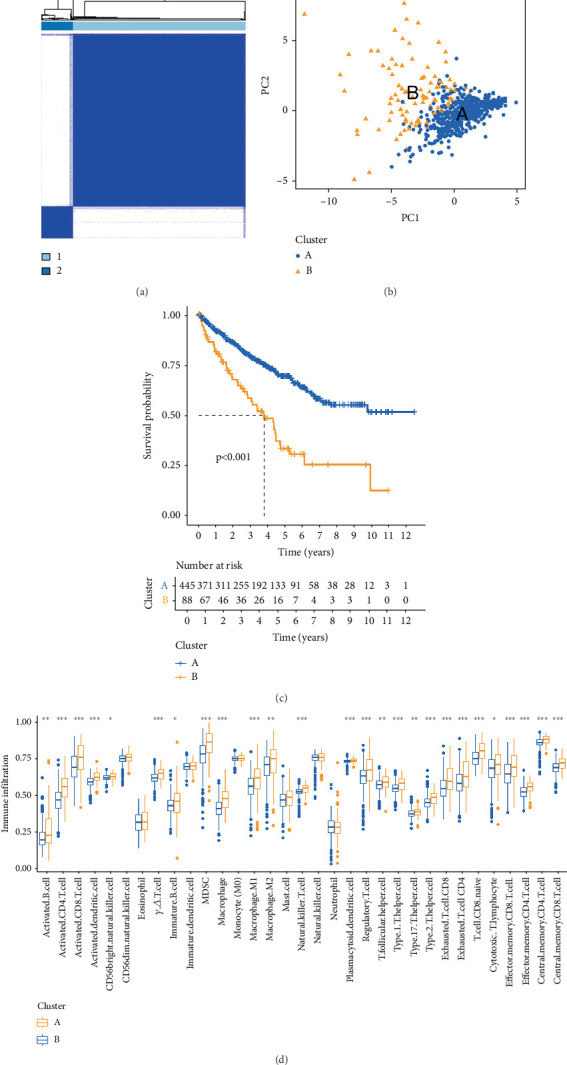
KIRC subtypes and their immune features. (a, b) Principal component analysis divided KIRC samples into two groups. (c) Survival analysis comparing high-risk and low-risk groups (*p* < 0.001). (d) Immune cell infiltration patterns between groups. ∗: *p* < 0.05; ∗∗: *p* < 0.01; ∗∗∗: *p* < 0.001.

RNA sequencing data and clinical information for 533 KIRC cases were acquired from the TCGA-KIRC dataset (https://portal.gdc.com). The R package ConsensusClusterPlus (v1.54.0) generated a matrix heatmap that differentiated samples by similarity, with high similarity indicated in blue and low similarity in white (Figure 2a). As illustrated, the distinction is clearest when *k* = 2, suggesting this is the optimal clustering number, representing minimal distribution inconsistency. Patients were therefore stratified into two stable groups via principal component analysis (Figure 2b), and OS and progression-free survival rates were compared between groups. Results showed that Group B demonstrated notably worse outcomes relative to Group A (*p* < 0.001) (Figure 2c), further underscoring the differential expression of candidate genes between the two groups.

Consensus clustering analysis divided KIRC samples into two groups, Group A and Group B, revealing marked variations in the expression of 15 prognostic factors between the groups. Single-sample gene set enrichment analysis (ssGSEA) was then executed to assess the correlation between these groups and immune cell populations, showing notable differences in immune cell expression. Our analysis revealed that although Group B exhibited a higher abundance of immune cell infiltration compared to Group A, with statistically significant overexpression of various CD4+ T cell and CD8+ T cell subsets, it still demonstrated a poorer prognosis. We hypothesize that this may be associated with the highly infiltrated immunosuppressive immune cells in Group B, including myeloid-derived suppressor cells (MDSCs) (*p* < 0.001), Tregs (*p* < 0.001), Th17 cells (*p* < 0.01), and M2 macrophages (*p* < 0.01), which may exert negative regulatory effects (Figure 2d).

### 3.3. Machine Learning and Nomogram Construction

**Figure 3:**
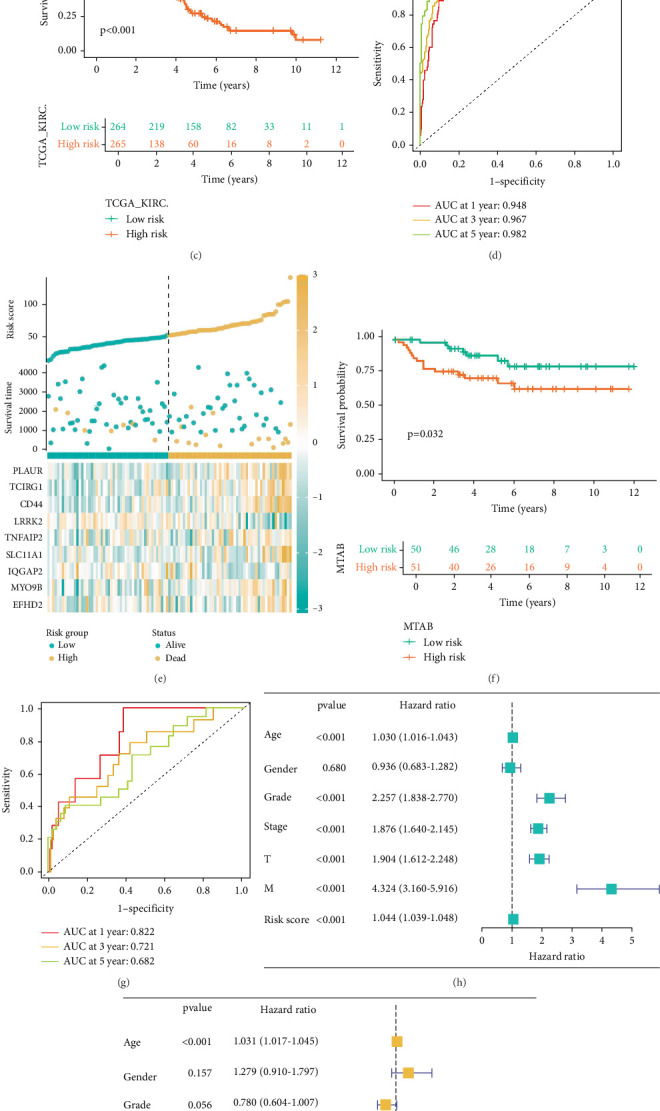
Construction of a prognostic model. (a) The optimal StepCox[both] + RSF algorithm combination was selected from 101 machine learning algorithm combinations to establish the prognostic model. (b) Risk scores of patients in the high-risk and low-risk groups within the training set. (c) Kaplan–Meier curves for the two patient groups in the training set. (d) Performance of the training set model in predicting 1-, 3-, and 5-year survival rates of KIRC patients. (e) Risk scores of patients in the high-risk and low-risk groups within the validation set. (f) Kaplan–Meier curves for the two patient groups in the validation set. (g) Performance of the validation set model in predicting 1-, 3-, and 5-year survival rates of KIRC patients. (h) Univariate Cox regression analysis. (i) Multivariate Cox regression analysis. (j) Construction of a nomogram based on risk scores and clinical characteristic parameters. (k) Calibration curves.

To predict the OS rate of patients with KIRC, we collected clinical datasets of KIRC patients from TCGA-KIRC. After excluding data with missing information, the remaining dataset was used as the training set (*n* = 529). The E-MTAB-1980 dataset from the ArrayExpress database (*n* = 101) served as the validation set. From 15 related genes, we further screened and identified nine core genes—TNFAIP2, TCIRG1, SLC11A1, PLAUR, MYO9B, LRRK2, IQGAP2, EFHD2, and CD44—using the StepCox[both] + RSF algorithm combination, which demonstrated the optimal *C*-index among 101 machine learning algorithm combinations (Figure 3a). These genes were used to construct a prognostic model. Patients were assigned a risk score (RiskScore) and divided into two groups: the high-risk group (top 50% of scores) and the low-risk group (bottom 50% of scores) (Figure 3b). The Kaplan–Meier (K-M) curve revealed that the survival rate of the high-risk group was significantly lower than that of the low-risk group (*p* < 0.001) (Figure 3c), indicating statistical significance. Similarly, in the validation set, patients were stratified based on the risk scores derived from the nine core genes (Figure 3e), and a statistically significant difference in survival rates between the high-risk and low-risk groups was observed (*p* = 0.032) (Figure 3f), confirming the model's validity. In terms of model evaluation, the AUC values for the training set at 1, 3, and 5 years were 0.948, 0.967, and 0.982, respectively (Figure 3d), while those for the validation set were 0.822, 0.721, and 0.682, respectively (Figure 3g), demonstrating strong predictive performance. Both univariate and multivariate Cox regression analyses confirmed that RiskScore is an independent risk factor (Figure 3h,i). To further enrich the model, we integrated RiskScore with clinical parameters such as age, gender, TNM stage, and pathological stage using the RMS package to generate a nomogram. This nomogram provided a comprehensive assessment of patient prognosis, with higher total scores indicating worse clinical outcomes (Figure 3j). The calibration curves for 1, 2, and 3 years showed good predictive accuracy (Figure 3k).

### 3.4. Key Gene TCIRG1 Identified

**Figure 4:**
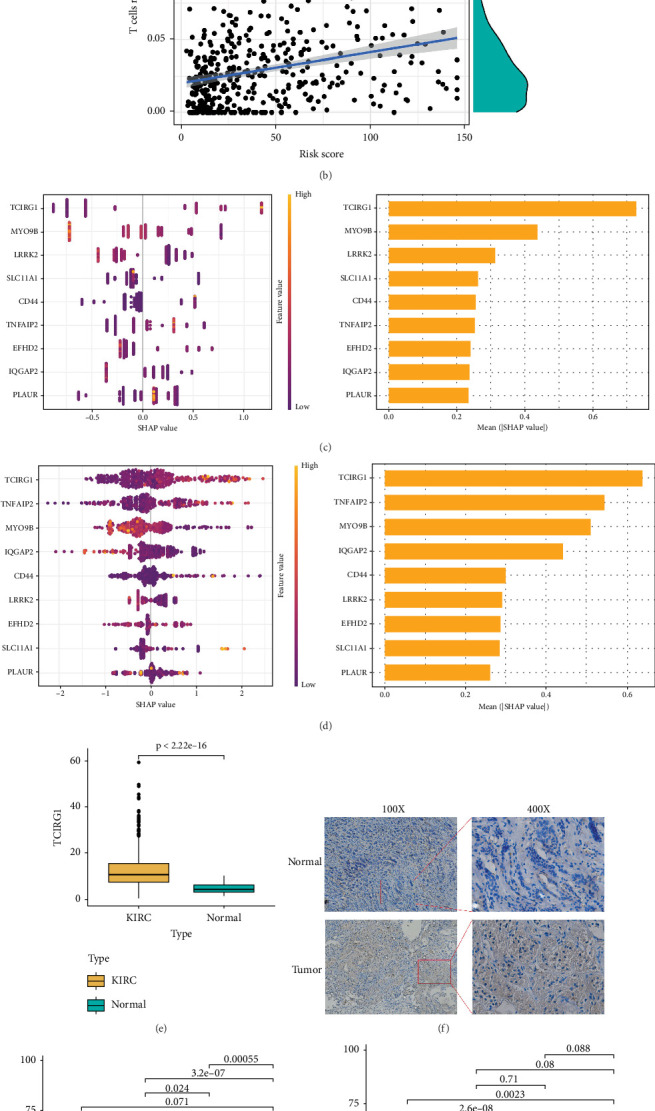
Screening of key gene and immune infiltration analysis. (a) Differences in immune cell infiltration abundance between high-risk and low-risk groups. (b) Correlation between Treg cells and RiskScore. (c) Machine learning validation with LightGBM and SHAP. (d) Machine learning validation with XGBoost and SHAP. (e) TCIRG1 expression differences between high-risk and low-risk groups. (f) Immunohistochemical staining of TCIRG1 in KIRC and normal kidney tissues. (g–j) Clinical characteristics of TCIRG1 expression.

Based on the expression differences of the feature genes set, we constructed a prognostic model and achieved risk stratification for KIRC patients. To further investigate the differences in immune cell infiltration within the TME between the high-risk and low-risk groups, we employed the CIBERSORT algorithm for TME analysis. The infiltration abundance of immune cells in the high- and low-risk groups was visualized. Violin plot results revealed statistically significant differences in the infiltration proportions of 11 immune cell populations between the high-risk and low-risk groups: T cells CD4 memory resting (*p* = 0.003), T cells CD4 memory activated (*p* = 0.010), T cells follicular helper (*p* = 0.003), Tregs (*p* < 0.001), monocytes (*p* = 0.017), macrophages M0 (*p* < 0.001), macrophages M1 (*p* = 0.003), macrophages M2 (*p* = 0.006), dendritic cells (DCs) resting (*p* = 0.020), mast cells resting (*p* < 0.001), and mast cells activated (*p* = 0.019) (Figure 4a). Scatter plot analysis demonstrated that Treg cells exhibited the strongest positive correlation with RiskScore, accompanied by the smallest *p* value (*p* = 2.4e − 11) (Figure 4b).

The TCGA database was used to establish training and validation sets, and the significance of the nine modeling genes was assessed through LightGBM and XGBoost machine learning (Figure 4c,d). SHAP values were then applied to interpret each gene's contribution to the model, revealing that TCIRG1 held the highest SHAP value in both machine learning analyses (Figure 4e), designating TCIRG1 as the central key gene with the most substantial impact on the model. Immunohistochemistry further validated the alternation of TCIRG1 expression in KIRC compared with the adjacent normal tissues. The staining results showed that the staining intensity of TCIRG1 in tumor tissues was significantly stronger than that in adjacent normal kidney tissues (Figure 4f). Analysis results confirmed TCIRG1 as the pivotal gene in our predictive model, showing distinct expression levels across KIRC samples of varying stages and characteristics (Figures 4g, 4h, 4i, and 4j).

### 3.5. Experimental Verification

**Figure 5:**
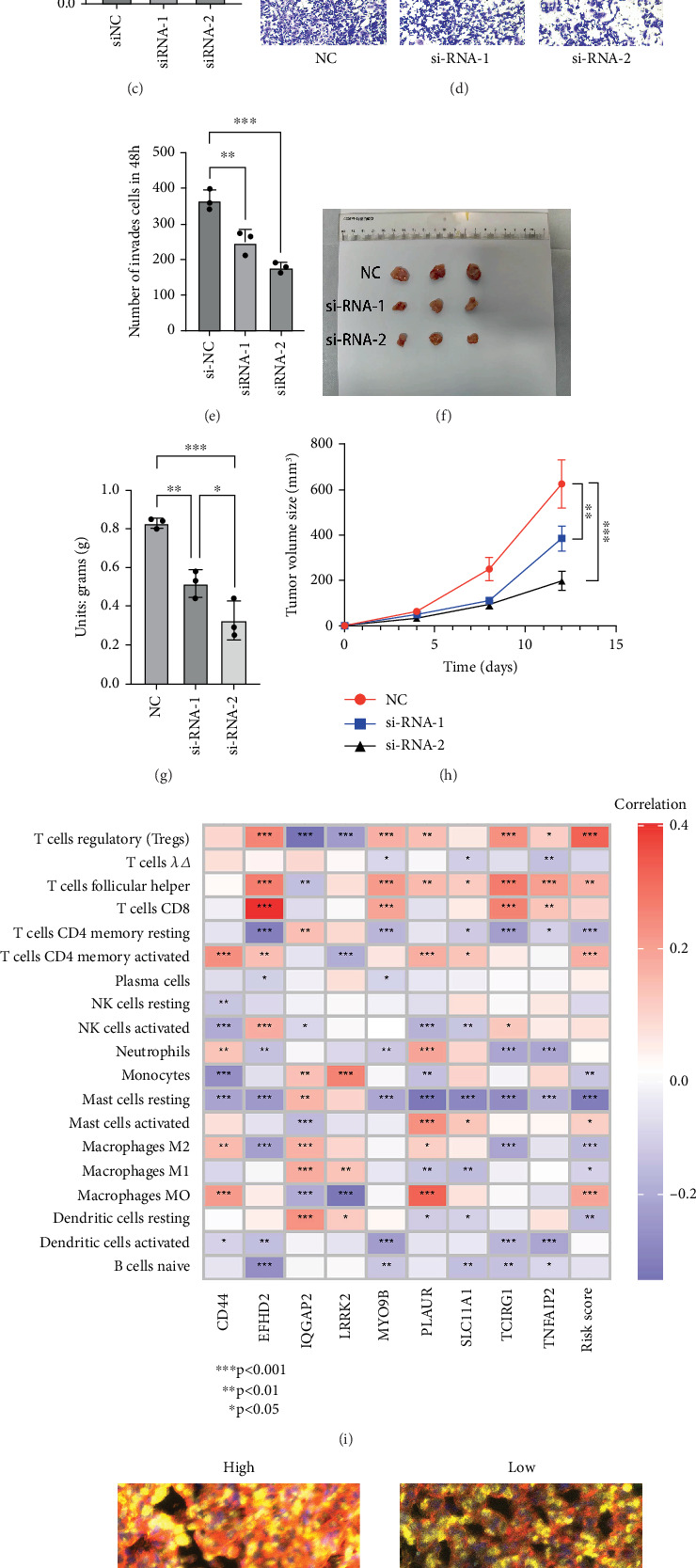
Expression and functional validation of the key gene TCIRG1. (a) TCIRG1 expression across different renal carcinoma cell lines. (b) TCIRG1 expression in 769-P cells 48 h post-siRNA transfection. (c) Knockdown histogram. (d) Transwell assay results showing the invasiveness of 769-P cells 48 h post-TCIRG1 knockdown, with invaded cell counts calculated using ImageJ software. All experiments were conducted in triplicate. (e) Transwell histogram. (f) Photographs of solid tumors in nude mice. (g) Tumor weight histogram. (h) Tumor volume statistical chart. (i) Immune coexpression analysis of prognostic factors. (j) Multiplex immunofluorescence staining demonstrates the presence of DAPI (blue), FoxP3 (green), CD152 (yellow), and TCIRG1 (red), along with control tissue showing minimal DAPI staining. ∗: *p* < 0.05; ∗∗: *p* < 0.01; ∗∗∗: *p* < 0.001.

To further explore the biological role of TCIRG1 in KIRC, we selected five different cell lines—HK-2, 786-O, 769-P, ACHN, and Caki-1—for subsequent experiments. Western blot results indicated that TCIRG1 expression was significantly higher in the 769-P cell line compared to normal renal tubular epithelial cells (HK-2) and other renal cancer cell lines (786-O, ACHN, and Caki-1) (Figure 5a). Therefore, we chose the 769-P cell line for further investigation. Western blot experiments confirmed the successful knockdown of TCIRG1 in the 769-P cell line (Figure 5b,c). Transwell assays demonstrated that the knockdown of TCIRG1 significantly reduced the invasive ability of 769-P cells (Figure 5d,e). After preliminary in vitro tissue and cellular experiments provided evidence of TCIRG1's protumorigenic role, we further designed a subcutaneous tumor formation experiment in nude mice to more intuitively demonstrate the promoting effect of TCIRG1 on KIRC growth in vivo (Figure 5f). The results showed that the weight (Figure 5g) and volume (Figure 5h) of transplanted tumors in the TCIRG1 knockdown group were significantly smaller than those in the control group.

TME analysis using the CIBERSORT algorithm revealed that Treg cells exhibited the strongest positive correlation with RiskScore (*p* < 0.001). Additionally, immune correlation heatmaps demonstrated a statistically significant positive correlation between Treg cells and the key gene TCIRG1 (*p* < 0.001), suggesting a potential association between TCIRG1 and Treg cells in KIRC, particularly in high-risk cases (Figure 5i). To further validate this, we conducted multiplex immunofluorescence experiments. Based on relevant literature, FoxP3 [36, 37] is recognized as the most sensitive and specific marker for activated Treg cells, while CD152 is also highly expressed on activated Treg cells. Using different fluorescent dyes to target these markers, multiplex immunofluorescence results showed a significant positive correlation between the fluorescence intensity of Treg cells and TCIRG1 across different renal cancer specimens, confirming their coinfiltration within the KIRC immune microenvironment (Figure 5j).

Based on the aforementioned data analysis and experimental results, we conclude that TCIRG1 is a key gene influencing the invasion and metastasis of KIRC. Additionally, it exhibits a significant correlation with immune cell infiltration within the KIRC TME, particularly with Treg cells.

## 4. Discussion

KIRC is the most prevalent and aggressive subtype of renal malignancies [7], garnering widespread attention in recent years for its diagnosis and treatment [12]. In the early stages of KIRC, partial nephrectomy can be performed to preserve more nephrons. However, if the tumor progresses to T2 stage or exhibits endophytic growth, more aggressive radical nephrectomy is often required. Unfortunately, early-stage KIRC often lacks obvious symptoms and signs, with initial diagnosis relying on imaging techniques such as ultrasound and CT. As a result, many patients are diagnosed at an advanced stage when they present with the classic triad of symptoms: flank pain, hematuria, and abdominal mass. Due to the resistance of KIRC to traditional radiotherapy and chemotherapy, the current mainstream treatment for these patients involves combination therapy targeting the immune microenvironment, such as ICIs and tyrosine kinase inhibitors (TKIs) [38]. Although immune-targeted therapies have shown some clinical efficacy, a significant proportion of patients develop resistance after 6–15 months of initial treatment. This resistance is attributed to the high heterogeneity and uniqueness of the immune microenvironment in KIRC, underscoring the urgent need to identify new and more suitable potential targets within the KIRC TME [12, 32].

Monocytes served as the entry point for our research. These cells exhibit strong migratory capabilities and can be recruited into the TME in response to damage-related chemokines such as CCL-2 and CXCL12 [39, 40]. Within the TME, monocytes differentiate into tumor-associated macrophages (TAMs), DCs, and MDSCs, primarily exerting immunosuppressive effects that correlate with poor patient prognosis. Among these, TAMs, under the stimulation of IL-4, IL-13, and TGF-*β*, predominantly exhibit M2-like macrophage characteristics. They release inhibitory cytokines such as IL-10 and TGF-*β* and express immune checkpoint molecules like PD-L1 to suppress T cell function. Additionally, TAMs can participate in glutamine metabolism, depleting nutrients essential for T cell activation. MDSCs promote tumor progression and metastasis by inducing epithelial–mesenchymal transition (EMT) and can also induce the generation of Tregs, exerting potent immunosuppressive effects within the TME. Our study initially revealed, through single-cell analysis, a significant enrichment of monocyte populations in the KIRC immune microenvironment compared to normal kidney tissue. Ultimately, we identified the key gene TCIRG1 from the subpopulations of this cell lineage and experimentally confirmed its association with Treg cells.

TCIRG1, also known as T cell immune regulator cDNA 7, encodes a protein that serves as a critical regulator of T cell immune responses, participating in the development, activation, and functional regulation of T cells. TCIRG1 is highly expressed in various malignancies, including prostate cancer, breast cancer, and hepatocellular carcinoma [31], and is involved in multiple pathways that promote tumor growth, invasion, and metastasis. A 2023 study by Li et al. [29] found that high expression of TCIRG1 in prostate cancer induces resistance to bicalutamide and accelerates progression to castration-resistant prostate cancer (CRPC). TCIRG1 is also implicated in the TME of KIRC, with its high expression predicting poor patient prognosis. A 2023 study by Di et al. [41] demonstrated that in KIRC, TCIRG1 functions as a core gene related to glycolysis, regulating aerobic glycolysis in KIRC through the AKT/mTOR pathway, thereby promoting malignant progression. Furthermore, TCIRG1 expression was significantly correlated with the infiltration of immune cells, particularly Th1 CD4+ T cells, CD8+ T cells, NK cells, and M1 macrophages. It also showed positive associations with PDCD1, CTLA4, and other immune checkpoints, as well as with CCL5, CXCR3, and additional chemokines and chemokine receptors. In our current study, immune analysis of the KIRC TME revealed a high degree of coinfiltration between TCIRG1 and Treg cells, a finding consistent with the gene's role in T cell immune regulation. Targeting or modulating the interaction pathways between these cells may represent a potential therapeutic strategy in the future.

Treg cells are a unique subset of CD4+ T cells with immunosuppressive functions [42–45]. Activated Treg cells highly and specifically express FoxP3, a transcription factor central to the development and function of Treg cells and a key target in our fluorescence staining experiments. Within the TME, Treg cells play a critical immunosuppressive role. Naive Treg cells express very low levels of FoxP3 upon exiting the thymus, but when recruited to inflammatory environments or the TME, they receive TCR stimulation from antigen-presenting cells and transform into highly immunosuppressive FoxP3(+) Treg cells. These cells exert their functions through multiple mechanisms. The IL-2 receptor on Treg cells, composed of *α* (CD25), *β* (CD122), and *γ* (CD132) subunits, has a much higher affinity for IL-2 than that of CD8+ T cells, giving Treg cells a competitive advantage in sequestering IL-2 within the TME. Since IL-2 is essential for the survival of CD8+ T cells, the depletion of IL-2 by Treg cells inhibits CD8+ T cell growth. Additionally, Treg cells secrete inhibitory cytokines such as TGF-*β*, IL-10, and IL-35, which downregulate the activation and proliferation of CD8+ T cells and induce the transformation of NK cells into type 1 innate lymphoid cells within the TME. These NK cells lack tumor-killing capabilities and instead promote immune tolerance. Furthermore, Treg cells secrete VEGF, promoting the formation of tumor-nourishing blood vessels and facilitating tumor invasion and metastasis. These findings demonstrate that Treg cells can alter the composition and activity of cells within the tumor TME through a complex network of molecular interactions, thereby influencing tumor progression.

In this study, we first collected the dataset (GSE139555) from the GEO database as the research subject. After quality control and filtering to remove low-quality cells, we performed single-cell analysis to compare immune infiltration between KIRC and normal kidney tissues. The results revealed that the proportion of monocytes increased most significantly in KIRC. Therefore, we further analyzed monocytes and extracted three subpopulations with the most significant enrichment differences. Combined with WGCNA, we identified 150 genes, and through differential expression analysis and Cox regression analysis using the TCGA-KIRC database, we screened out 15 KIRC-related genes. Based on these 15 genes, we performed consensus clustering analysis and divided KIRC patients into Group A and Group B, with Group B showing significantly worse prognosis and distinct immune infiltration patterns compared to Group A. Using the TCGA-KIRC dataset as the training set (*n* = 529) and the ArrayExpress E-MTAB-1980 dataset as the validation set (*n* = 101), we evaluated 101 machine learning algorithm combinations and selected the optimal StepCox[both] + RSF algorithm combination. This process identified nine core genes—TNFAIP2, TCIRG1, SLC11A1, PLAUR, MYO9B, LRRK2, IQGAP2, EFHD2, and CD44—to construct a prognostic model for KIRC. Survival analysis demonstrated significant differences in survival time between high-risk and low-risk groups in both the training and validation sets, with high AUC values, indicating robust predictive performance. A nomogram incorporating clinical parameters further confirmed the model's reliability. By using the CIBERSORT algorithm, we analyzed the composition of major immune cells in the KIRC TME, revealing that Treg cells exhibited the most significant expression differences between the risk groups. Subsequently, we employed LightGBM and XGBoost machine learning algorithms to identify TCIRG1 as the key gene with the highest contribution to the model. Through clinical data analysis, immunohistochemistry, and western blot, we confirmed the differential expression of TCIRG1 in normal kidney tissues and KIRC. Transwell assays and subcutaneous tumor formation experiments in nude mice demonstrated that tumor tissues with high TCIRG1 expression exhibited stronger invasiveness. Finally, multiplex immunofluorescence validated the coinfiltration of TCIRG1 and Treg cells. In conclusion, our prognostic model, along with the validation of the biological functions of the key gene and immune infiltration, provides a new research direction for the treatment of KIRC.

This study has certain limitations. While TCIRG1 and Treg cell coexpression in renal carcinoma cells was confirmed, the precise mechanisms and pathways driving tumor growth, invasion, and metastasis remain insufficiently explored. Further in vivo studies are necessary to validate TCIRG1's tumor-promoting effects. Additionally, bioinformatic analysis suggests the involvement of other immune cells in KIRC progression. Future studies will involve more extensive clinical and foundational research to strengthen the basis of our current findings.

## 5. Conclusion

This study developed a novel predictive model to assess KIRC prognosis, integrating single-cell analysis and RNA data, and validated the biomarker TCIRG1, which is strongly associated with KIRC progression and prognosis. TCIRG1 expression was markedly elevated in KIRC tissues and correlated with shorter OS. Furthermore, TCIRG1 expression showed a strong association with infiltrating immune cells, particularly CD4+ Th1 cells, CD8+ T cells, NK cells, and M1 macrophages. mIHC confirmed the link between TCIRG1 and Treg immune infiltration, offering a promising new avenue for KIRC immunotherapy.

## Data Availability

The data that support the findings of this study are available from the corresponding authors upon reasonable request.
